# Structural connectivity associated with the sense of body ownership: a diffusion tensor imaging and disconnection study in patients with bodily awareness disorder

**DOI:** 10.1093/braincomms/fcac032

**Published:** 2022-02-11

**Authors:** Antonino Errante, Alice Rossi Sebastiano, Settimio Ziccarelli, Valentina Bruno, Stefano Rozzi, Lorenzo Pia, Leonardo Fogassi, Francesca Garbarini

**Affiliations:** 1 Department of Medicine and Surgery, University of Parma, Parma 43125, Italy; 2 MANIBUS Lab, Psychology Department, University of Turin, Turin 10123, Italy; 3 SAMBA Research Group, Psychology Department, University of Turin, Turin 10123, Italy; 4 Neuroscience Institute of Turin (NIT), Turin 10123, Italy

**Keywords:** body ownership, pathological embodiment, diffusion tensor imaging, ventral premotor cortex, posterior parietal cortex

## Abstract

The brain mechanisms underlying the emergence of a normal sense of body ownership can be investigated starting from pathological conditions in which body awareness is selectively impaired. Here, we focused on pathological embodiment, a body ownership disturbance observed in brain-damaged patients who misidentify other people’s limbs as their own. We investigated whether such body ownership disturbance can be classified as a disconnection syndrome, using three different approaches based on diffusion tensor imaging: (i) reconstruction of disconnectome maps in a large sample (*N* = 70) of stroke patients with and without pathological embodiment; (ii) probabilistic tractography, performed on the age-matched healthy controls (*N* = 16), to trace cortical connections potentially interrupted in patients with pathological embodiment and spared in patients without this pathological condition; (iii) probabilistic ‘*in vivo*’ tractography on two patients without and one patient with pathological embodiment. The converging results revealed the arcuate fasciculus and the third branch of the superior longitudinal fasciculus as mainly involved fibre tracts in patients showing pathological embodiment, suggesting that this condition could be related to the disconnection between frontal, parietal and temporal areas. This evidence raises the possibility of a ventral self-body recognition route including regions where visual (computed in occipito-temporal areas) and sensorimotor (stored in premotor and parietal areas) body representations are integrated, giving rise to a normal sense of body ownership.

## Teaser

Errante *et al.*’s study demonstrates that pathological embodiment, a body ownership disturbance observed in stroke patients who misidentify other people’s limbs as their own, emerges from the disconnection of multiple temporal, parietal and premotor areas, which play a key role in integrating visual and sensorimotor body representations.

## Graphical Abstract

**Graphical Abstract:**
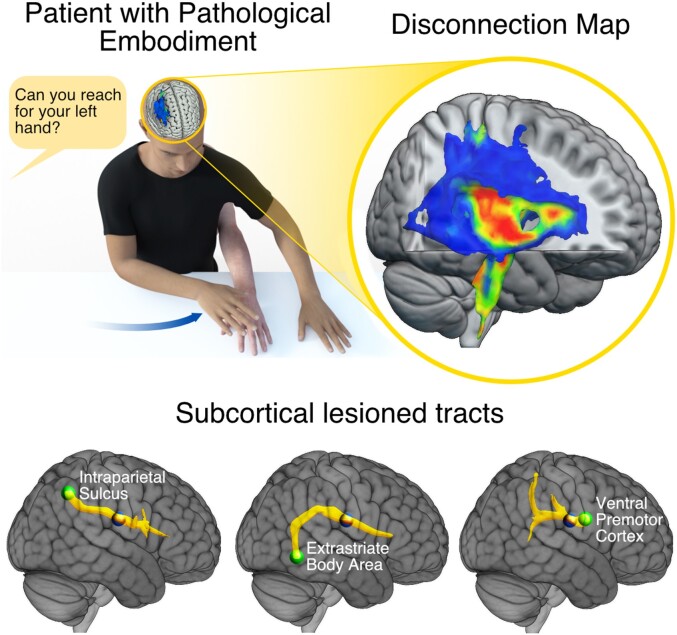

## Introduction

A challenge for neuroscience is to understand the neural process underlying the construction of human self-awareness. A fundamental component of our self-experience is the body we live in, the mean by which we perceive and act. What makes bodily sensations so unique is the feeling that ‘my body’ belongs to me and is ever present in my mental life; i.e. the so-called sense of body ownership.^1^ How does this sense of body ownership emerge from our brain? A critical contribution for understanding the anatomical substrate of human body awareness comes from a neuropsychological perspective that uses pathological conditions, where the body awareness is selectively impaired, to make inferences about the brain processing underlying the emergence of a normal sense of body ownership.

We focused on a monothematic delusion of body ownership that has been observed in patients with a diagnosis of ischaemic or haemorrhagic stroke who do not explicitly deny the ownership of their contralesional limbs (as in somatoparaphrenia^2^), but, conversely, misidentify other people’s limbs as their own.^3,4^ To refer to this clinical manifestation, the term pathological embodiment (PE) has been coined.^5^ Stroke patients who are affected by PE are classified as E+ (whereas those not affected are E−). Whenever the examiner’s hand is in a body-congruent position (i.e. aligned with the patient’s shoulder and perceived from a first-person perspective), E+ patients claim that this ‘alien’ hand is their own, and they treat and care for it as if it were their own hand. In previous studies, it has been demonstrated that PE is not a mere verbal confabulation, since the alien (embodied) hand is functionally treated by the neural system as the patient’s own hand, in both motor^5–7^ and sensory domains.^8,9^ From an anatomical point of view, previous evidence based on standard lesion mapping in patients affected by a body ownership disturbance, such as somatoparaphrenia^10^ and PE,^9,11^ suggests that these deficits could be mainly associated with subcortical lesions.

Here, we asked whether PE can be classified as a disconnection deficit and we aimed to identify the fibre tracts that are mainly involved in this pathological condition, to propose a neural mechanism for the construction of the sense of body ownership based on neuropsychological data. To this aim, we took advantage of the advanced structural connectivity analyses on a large cohort of patients as it has already been successfully reported by a previous study investigating neural underpinnings of neuropsychological deficits at group level.^12–14^ Here, we compared E+ and E− patients, by combining three different approaches based on the reconstruction of disconnection maps and diffusion tensor imaging (DTI) probabilistic tractography.

In order to trace cortical connections of fibre bundles that are potentially interrupted in patients, we ran two different analyses. In the first analysis, starting from lesion maps in a large sample (*N* = 70) of stroke patients with and without PE, we quantified the probability of disconnection of a series of subcortical bundles included in a template of 10 healthy controls.^15^ In the second analysis, in order to restrain the tract reconstruction within the body ownership network, we collected DTI data in 16 age-matched healthy subjects and performed probabilistic DTI tractography by using body ownership-related cortical regions of interest (ROIs) as seeds and the lesion overlapping of E+ and E− as waypoints. As for the ROIs, they were selected within sensory-motor areas previously linked to the sense of body ownership (i.e. ventral premotor cortex, PMv and intraparietal sulcus, IPS^16^), and high order visual areas involved in the visual representation of the body (i.e. extrastriate body area, EBA^17^). As for the seeds, we identified the regions more frequently damaged in E+ and E− patients, respectively, using lesions overlap. This allowed to localize two subcortical regions in the deep with matter, one for E+ group and one for E− ones. Thus, we entered these two regions in the DTI tractography to reconstruct the lesioned fibre bundles in the two groups separately, originating from body ownership-related ROIs.

In the third analysis, to directly measure which fibre bundles were actually interrupted in representative cases, we selected three further patients (two E− and one E+) to collect DTI sequences and to conduct tractography analysis.

We expected that this combined approach might contribute to the understanding of the paradoxical behaviour shown by E+ patients, who misidentify other people’s hand as their own, also providing new perspectives to make inferences about the normal functioning of brain networks underlying the emergence of the sense of body ownership.

## Materials and methods

### Patients

**Figure 1:**
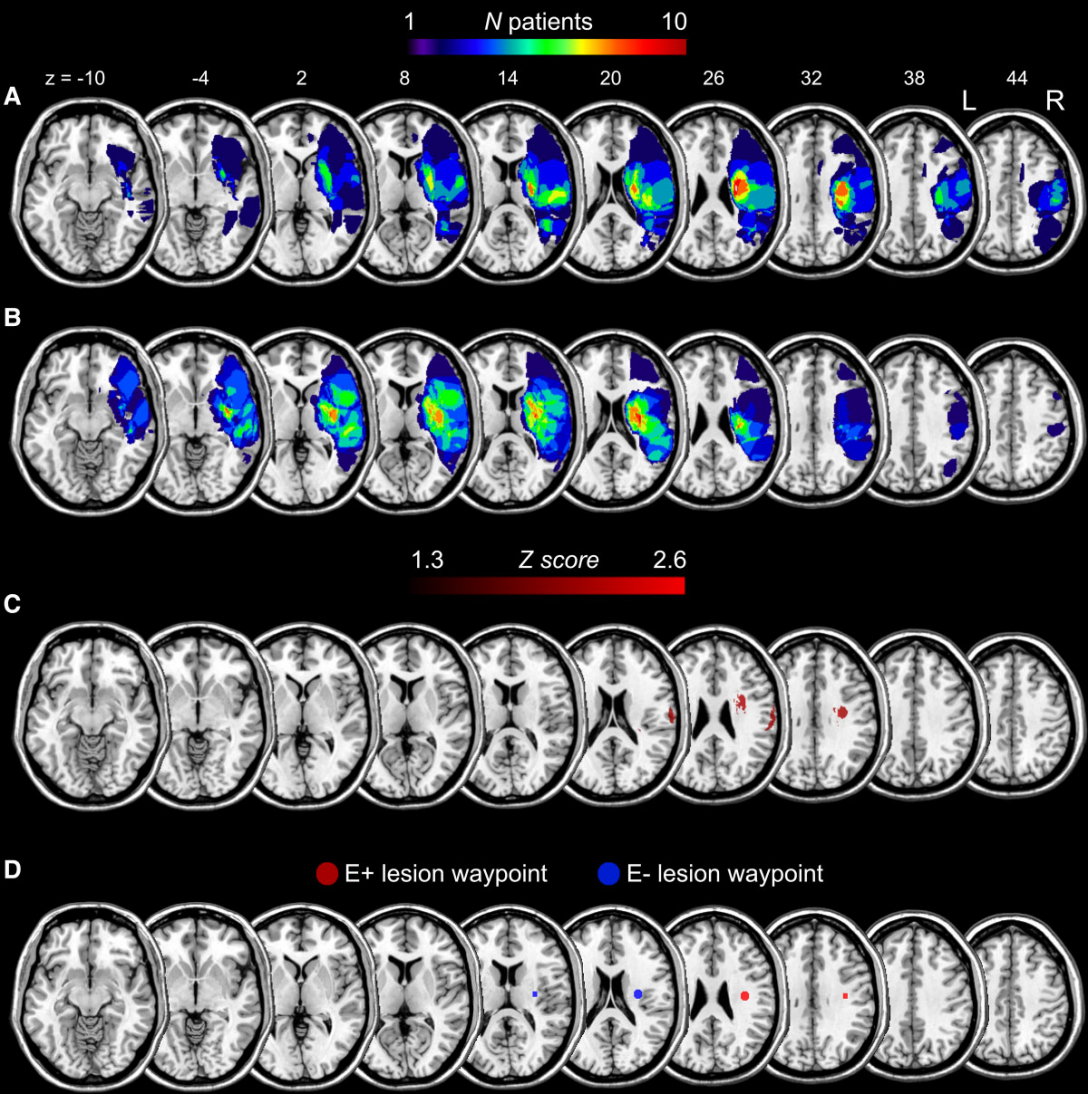
**Lesion topography.** Lesion overlap topography of the restricted group of 16 E+ (**A**) and of 16 E− (**B**) patients balanced for secondary deficits. The colour bars indicate the number of overlapping lesions. Numbers above the slices indicate *z*-coordinates in MNI space. (**C**) Brain regions are significantly associated with the E+ group. All voxels which survived to the binomial test (*P* < 0.01, uncorrected) are displayed. The colour scale represents Z-Libermeister scores. (**D**) Visualization of the position of E+ lesion waypoint (red coloured sphere), corresponding the maximum lesion overlap in E+ patients (MNI, *x* = +36, *y* = −14, *z* = 28).

Lesion data (Fig. 1A and B; Supplementary Fig. 2) were collected from the database (Department of Psychology of University of Turin) of consecutive stroke patients in the sub-acute or chronic phase admitted to several rehabilitation units from 2010 to 2018. The inclusion criteria to be enrolled in the present investigation were the presence of unilateral acquired brain damage, contralesional upper limb motor impairment and/or sensory deficits, absence of aphasia or other language deficits or previous neurological disease. Accordingly, 70 brain-damaged patients (see Supplementary Table 1) aged between 37 and 85 years (66.4 ± 10.2 years; 31 females 39 males) were included. In particular, patients were screened with the Italian version of Mini Mental State Examination (MMSE)^18^ and/or the Montreal Cognitive Assessment^19^ in order to evaluate the overall cognitive impairment. Contralesional motor and tactile deficits were also assessed according to the standardized protocol of Bisiach and Faglioni,^20^ whereas proprioception was assessed by means of Joint Position Matching Task.^21^ Awareness, for tactile and motor deficit, was assessed according to the previously published procedure.^22,23^ The presence of left extrapersonal neglect was assessed with Behavioural Inattention Test,^24^ while left personal neglect with the Bisiach methodology.^25^ Further assessment investigated the presence of somatoparaphrenia.^26^ The patients were assessed for the presence/absence of PE using behavioural procedures^11^ in which the patient had to identify her/his own hand when the examiner’s arm was simultaneously presented near the patient’s arm. Neurological and neuropsychological evaluations are reported in Supplementary Fig. 1 and Table 1. Accordingly, patients were divided into two groups: a group of 35 E+ patients aged between 45 and 85 years (69.9 ± 9.1 years; 17 females, 18 males), and a group of 35 E− patients aged between 37 and 83 years (62.9 ± 10.3 years; 14 females, 21 males). To test whether the two groups differed in motor, tactile and proprioceptive impairment, as well as extrapersonal neglect, between-group statistical comparisons were performed by means of two-sided tests on the proportion of impaired patients. Results revealed that the two groups were matched in terms of motor (*P* = 0.27) and tactile (*P* = 0.62) deficits, whereas the E+ group showed a significantly higher proportion of patients with proprioceptive impairment (*P* = 0.0002) and extrapersonal neglect (*P* = 0.0003) relative to the E− group. Hence, we selected from this sample a subset of 32 patients (E+: *N* = 16, E−: *N* = 16) accurately matched so that the two groups did not differ in terms of motor (*P* = 1.0), tactile (*P* = 0.74) and proprioceptive (*P* = 0.19) primary deficits, as well as extrapersonal neglect (*P* = 0.63) (Supplementary Fig. 1). The study was approved by the Ethical Committee of the ASLTO1 of Turin (N:46485/13), all patients gave written, informed consent and the research was conducted in accordance with guidelines of the Declaration of Helsinki. The manuscript was prepared according with the STROBE checklist for case–control studies.

### MRI data acquisition

Magnetic resonance data were acquired using a 3-T MR scanner (GE Discovery MR750) at Parma University Hospital. The images were collected with a dedicated 32-channel head-coil using the following protocol. A high-resolution 3D isotropic T_1_-weighted-images sequence (called BRAVO, BRA in volume) was acquired as an anatomical reference, and consisted in a 3D inversion recovery-prepared, fast spoiled gradient-echo recalled (IR FSPGR) sequence: 0.9 mm × 0.9 mm × 0.9 mm, inversion time (TI) = 650 ms, echo time (TE) = 4 ms, repetition time (TR) = 9.7 ms, flip angle (FA) = 9°, MATRIX = 512 × 512 × 192, receiver bandwidth (BW) = 98 Hz/pixel; a DTI spin-echo single-shot echo-planar imaging (EPI) sequence, with TR/TE 56586/82.4 ms, 2 mm isotropic voxels, 64 encoding directions with an effective *b* value of 1000 s/mm^2^, eight images with no diffusion weight in anterior–posterior phase encoding direction and other eight images with no diffusion weight in the reverse phase encoding direction, was added to the MR protocol in order to investigate the white matter connectivity.

### Lesion topography

Ischaemic lesion’s location was identified by means of MRI or CT scans and registered to the ICBM152 template of the Montreal Neurological Institute (MNI), included with the MRIcron software (ch2better; https://www.nitrc.org/projects/mricron). The lesions drawing was performed blindly and independently by two expert neuroradiologists, prior (blind) to the group classification, in cases of disagreement of a lesion drawing a third anatomist was consulted (<5% of cases). As the two patients have brain lesions on the left side of the brain, the images of these subjects were flipped so that all lesions are shown as right-side images. Importantly, the extent of the lesion volume did not differ between groups both in the large (independent pairs *t*-test: *t*_69_ = 0.87; *P* = 0.39) and in the restricted (*t*_31_ = 0.15; *P* = 0.88) sample. For visualization of lesion overlap in the restricted groups, see Fig. 1A and B. Lesion overlap in the large group of patients is shown in Supplementary Fig. 2.

Univariate voxel-based lesion symptom mapping (VLSM) was carried out using the NPM tool included in MRIcron (https://www.nitrc.org/projects/mricron) to identify the most damaged voxels in patients with (E+) as compared with patients without (E−) PE (see also section ‘Statistical analyses’). Figure 1C represents the results of the voxel-by-voxel test and shows a lesional cluster for the E+ group centred on the superior longitudinal fasciculus (SLF). This region, largely corresponding to the maximum lesion overlap in E+ patients (Fig. 1A), was used in the subsequent tractography analysis as waypoint mask (Fig. 1D) in order to restrain tracts reconstruction (see section ‘Definition of cortical seed regions and waypoints for probabilistic tractography’ for methodological details about masks definition).

### Disconnectome maps

Disconnectome maps were computed by using the ‘Disconnectome Map’ tool included in the BCBToolkit software (http://toolkit.bcblab.com).^27^ This approach uses a set of 10 healthy controls^15^ diffusion-weighted datasets to track fibres passing through each patient lesion. In particular, each lesion in MNI space was registered to each control (native space) using affine and diffeomorphic transformations^28,29^ and subsequently used as the seed for tractography in Trackvis.^30^ Thus, tractography from the lesions was transformed in visitation maps,^31^ binarized and remapped to MNI space using the inverse of precedent deformations. Finally, we produced a percentage overlap map for each group of patients summing at each point in MNI space the normalized visitation map of each healthy subject. In the resulting disconnectome map, the value of each voxel represents the inter-individual variability of tract reconstruction in controls, indicating the probability of disconnection to 0–100% for the given lesion.^32^

To quantify the severity of the disconnection in each group of patients, ‘Tractron’ tool included in the BCBToolkit (http://toolkit.bcblab.com) was used. This tool allows computing the probability (0–100%) that each patient’s lesion spatially corresponds to a specific white matter tract. In more detail, to estimate this correspondence, each patient’s lesion is compared with an atlas of white matter tracts,^15^ thus indicating the probability of matching between the lesion and tract atlas [such as, for example, arcuate fasciculus (AF), SLF or corticospinal (CS) tract] in the MNI152 coordinate system. Subsequently, disconnection probabilities as computed by Tractron, for each patient, were used for statistical group analysis. We ran a group-based disconnection analysis including the subgroup (16 E+ versus 16 E−) of patients matched for motor, tactile and proprioceptive deficits, as well as extrapersonal neglect. In order to highlight significant differences between the two groups of patients, the percentages of tract disconnection were entered, as dependent variables, a one-way MANOVA (see section ‘Statistical analyses’). In this analysis, individual percentages of disconnection for 10 specific tracts well described in the literature (arcuate anterior, arcuate long, arcuate posterior, corpus callosum, corticospinal, fronto-striatal, inferior fronto-occipital, SLF I, SLF II, SLF III) were entered as the dependent variables while *Group* (E+ and E−) was considered as between-subject factor.

For completeness, we carried out the same analysis also on the large cohort of patients not matched for secondary deficits. The results of this analysis are reported in Supplementary Figs 3 and 4.

In order to verify which disconnected tract discriminates the two groups in the restricted cohort of patients (balanced for secondary deficits), a binary probit regression was carried out, using the STATISTICA StatSoft software (https://www.statistica.com/en/).

### Probabilistic diffusion tractography

#### DTI dataset

Scans were obtained from the 16 age-matched healthy subjects (nine females, all right-handed, average age 59 ± 6 years). None of the subjects had a history of neurological or psychiatric disease and all were screened for motor and perceptual deficits. The local ethics committee of the University of Parma (Protocol number: UNIPRMR750v1) approved the study and all participants gave their written informed consent.

#### Case series study

To further support the probabilistic tractography reconstruction based on DTI, performed on healthy subjects, we collected DTI data of three brain-damaged patients not belonging to the main group of 70 patients and performed ‘*in vivo*’ tractography. One of them had no signs of neglect (N−) nor presence of delusion of ownership (E−) (Patient #1), another one had signs of neglect (N+) but no signs of delusion of ownership (E−) (Patient #2), and the remaining one showed both neglect and PE (N+E+; Patient #3) (detailed clinical data are reported in Supplementary Table 1).

#### Processing of DTI data

All DTI data processing was performed offline using the FMRIB Software Library (FSL) tools (version 5.0.9),^33–35^ with a dedicated workstation. Source images were corrected for head motion and distortions caused by eddy currents and difference in the susceptibility distribution in the brain with the combination of the FSL’s tools TopUp^33,36^ and Eddy^37^ using the data collected with reversed-phase encoding directions. BET tool was used for brain extraction using as input both T_1_ and the averaged B0 images.^38^ FMRIB’s Linear Image Registration Tool (FLIRT) was used for linear (affine) intra- and inter-modal image registration to MNI152 template (2 mm resolution).^39^

#### Definition of cortical seed regions and waypoints for probabilistic tractography

The cortical seed regions for probabilistic fibre tracking were localized on the basis of previous functional investigations showing the involvement of posterior parietal cortex, premotor cortex and occipito-temporal high order visual areas in building the sense of body ownership.^16,40–45^ In particular, the IPS (*x* = +30, *y* = −56, *z* = +52) and the PMv (*x* = +48, *y* = −2, *z* = +28) seeds have been localized on the basis of a previous meta-analysis of fMRI studies on body ownership.^16^ The EBA (*x* = +50, *y* = −62, *z* = +4) seed was identified on the basis of previous fMRI studies on body representation in the posterior temporal lobe.^40,46,47^ The three cortical seeds (sphere with a radius of 5 mm) were used in the subsequent probabilistic tractography in order to obtain maps of structural white matter connectivity representing the most probable connections starting from each seed (IPS, PMv, EBA). In the resulting maps, each voxel value indicates the total number of streamlines crossing that voxel.

Because cortical ROIs included temporal, parietal and frontal areas largely and reciprocally interconnected, we used a complementary method provided by the FSL software (named ‘waypoint mask’) to filter out those streamlines not relevant for the analysis (e.g. streamlines not part of white matter tracts passing through the patients’ lesions). In order to select the most frequently damaged territories in the two cohorts of patients matched for clinical features (16 E+ and 16 E−, respectively), we started from the results of lesions topography (see Fig. 1). We specifically looked at the contribution of subcortical lesions because the maximum lesion overlap resulting from this analysis (>65%) was found in the deep white matter for both groups of patients. Specifically, the centre of these overlap regions was at the following MNI coordinates: E+ lesion overlap, *x* = +36, *y* = −14, *z* = +28; E− lesion overlap, *x* = +30, *y* = −12, *z* = +18. Note also that E+ maximum lesion overlap (Fig. 1D) largely corresponds to the voxels identified as more frequently damaged in E+ patients using VLSM (Fig. 1C) (see section ‘Lesion topography’). Therefore, we created two spherical waypoint masks, one for E+ patients’ lesion, and one for E−, centred at these coordinates and included them in the tractography reconstruction. As a result, only streamlines originating from the cortical seed ROIs and passing through the waypoint masks have been reconstructed. Both masks were then resliced to the native space of each healthy subject’s DTI data, and enlarged to a sphere with a radius of 5 mm.

#### Probabilistic tractography estimation

Estimation of tracts was done using published methods in the FSL environment.^48^ Fibre tracking was done probabilistically, using 5000 tract-following samples at each voxel. We used a dual-fibre model as implemented in the latest version of BEDPOSTX tool, part of the FDT software package.^48,49^ This model helps to account for issues related to crossing fibres and produces more reliable results compared with the single-fibre models. Tractography analyses were performed using PROBTRACKX, a probabilistic algorithm that samples from Bayesian distributions of multiple diffusion directions to facilitate tracking through crossing fibres.

This results in a brain image in which all voxels have a value that represents the connectivity (number of fibres from the probabilistic analysis) between that voxel and the voxels in the seed region (i.e. IPS, PMv or EBA). One advantage of the probabilistic tractography is that it accounts for uncertainty inherent in the local fibre directions, and thus estimates a spatial probability distribution of connectivity from the seed regions. In our analysis, we used also an additional waypoint to restrain the tractography. Therefore, if a particular streamline did not pass through the waypoint mask, it was rejected.

All tractography analyses were done in each subject’s native space. All the result images were visually inspected to ensure correct normalization and tracking. The resulting statistical maps were then thresholded at 0.01 (99% likelihood of connectivity) and their values normalized to a range of 0–100.000 and transferred into MNI 152 1 mm standard space for cross-subject analysis. To generate population-based probability maps, all subjects’ significant maps (after thresholding), reconstructed from the same seed, were combined. A mean statistically significant tract for each seed was calculated by adding up all single-subject tracts and calculating a mean image using the MRIcron software.

In order to highlight the most involved tracts in E+ versus E− patients, we first averaged, separately, the three normalized and statistically thresholded tracts referred to E+ (Tract IPS to E+ lesion waypoint AND Tract EBA to E+ lesion waypoint AND Tract PMv to E+ lesion waypoint), and the three bundles referred to E− (Tract IPS to E− lesion waypoint AND Tract EBA to E− lesion waypoint AND Tract PMv to E− lesion waypoint), resulting in one E+ mean lesioned tract and one E− mean lesioned tract. The resulting mean tracts were statistically significant and normalized. For illustrative purposes, we computed also a pairwise subtraction using the SPM toolbox ImCalc (https://www.nitrc.org/projects/imcalc/), showing the difference between the two averaged tracts (E+ versus E−).

#### Tractography reconstruction in three patients

DTI based tractography was performed on three further patients, two E− and one E+ (see Supplementary Table 1), to reconstruct in both hemispheres the CS tract, the three branches of the SLF and the AF (posterior and long segment) following the procedure provided by the human brain atlases.^50,51^ Descending fibres of CS from the fronto-parietal cortex to subcortical nuclei and spinal cord were traced using a single seed ROI (5 mm spheres; LH, *x* = −22, *y* = −10, *z* = +21; RH, *x* = +20, *y* = −10, *z* = +21) localized around the anterior and posterior arms of the internal capsule.

ROIs (5 mm radius) for the dissection of SLF were created starting from the anatomical reference (SPM Anatomy toolbox v1.7)^52^ including:

- Inferior frontal gyrus (IFG) (LH, *x* = −53, *y* = +7, *z* = +22; RH, *x* = +52, *y* = +7, *z* = +22) that also includes the PMv;
- Dorsal sector of premotor cortex (PMd) (LH, *x* = −26, *y* = −8, *z* = +60; RH, *x* = +28, *y* = −8, *z* = +62);
- Inferior parietal lobule (IPL) (LH, *x* = −58, *y* = −44, *z* = +40; RH, *x* = +60, *y* = −44, *z* = +40);
- IPS (LH, *x* = −32, *y* = −59, *z* = +51; RH, *x* = +30, *y* = −58, *z* = +50), labelled as Areas hIP2/hIP3.

The SLF I was reconstructed as the tract that connects the PMd with SPL. SLF II was identified as the tract connecting the PMv with the posterior inferior parietal cortex. SLF III was defined as the tract that connects the PMv and the IFG with the rostral inferior parietal cortex.

A two-ROIs approach was used to perform a detailed dissection of the AF (ROI1, LH, *x* = −34, *y* = −44, *z* = +36, RH, *x* = +34, *y* = −44, *z* = +36; ROI2, LH, *x* = −34, *y* = −18, *z* = +32, RH, *x* = +34, *y* = −18, *z* = +32), allowing to include different sets of fibres within the arcuate bundle. These bundles include the classical large half-moon shaped region on the dorsal most part of the AF, and a lower region connecting posterior temporal and inferior parietal areas.

In order to exclude interhemispheric connections between seeds, we used a middle-sagittal exclusion mask.

### Statistical analyses

In order to test whether the two groups differed in secondary deficits, i.e. motor, tactile and proprioceptive impairment, as well as extrapersonal neglect, between-group statistical comparisons were performed by means of independent pairs *t*-test on the proportion of impaired patients. The same statistical test was used also to compare total lesion volumes in the two groups of patients. Statistical threshold was set at one-tailed *P* < 0.05. Voxel-wise data obtained from VLSM were analyzed using Liebermeister test for binary data, with a threshold set at one-tailed *P* < 0.01 (uncorrected) as implemented in the NPM tool, included in the MRIcron (https://www.nitrc.org/projects/mricron). In order to highlight significant differences in tracts disconnection between E+ and E− groups of patients, the percentages of tract disconnection were entered, as dependent variables, a one-way MANOVA. In this analysis, individual percentages of disconnection for the selected tracts were entered as dependent variables while *Group* (E+ and E−) was considered as between-subject factor. The statistical threshold for this analysis was set at *P* = 0.05. *Post hoc* comparisons based on paired-sample *t*-tests, with Bonferroni correction were used to assess significant differences.

### Data availability statement

The data that support the findings of this study are available from the corresponding author, upon reasonable request.

## Results

### Disconnection results

**Figure 2:**
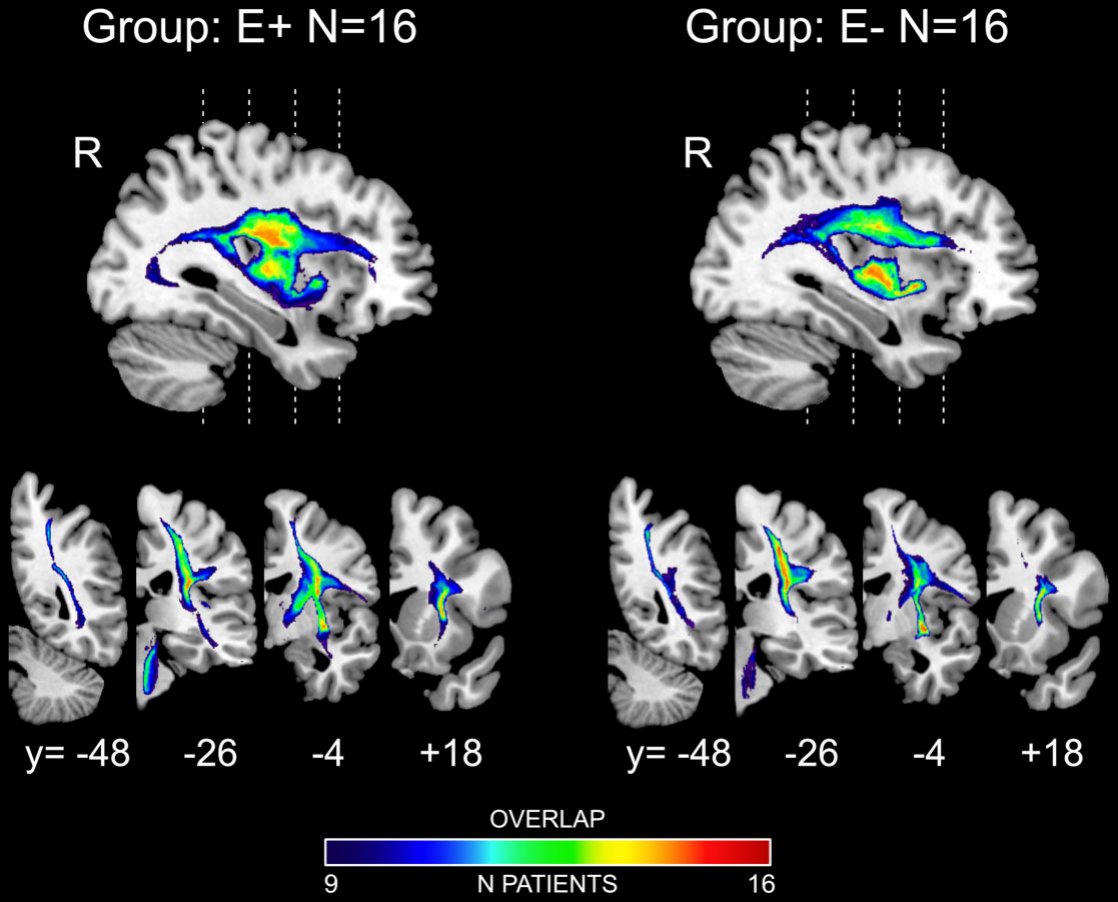
**Disconnectome maps.** Disconnectome maps resulting from individual lesions averaged across the restricted cohort of E+ (*N* = 16) and E− (*N* = 16) patients. Each map is overlaid on MNI template and presented in one medial parasagittal (*x* = +36 in MNI space), and four representative right coronal sections (numbers indicate *y*-coordinates in MNI space). The colour bars indicate the number of overlapping disconnectome maps.

To map the disconnection of white matter fibre bundles in E+ and E− patients, we used each patient’s lesion map to create a probability map of disconnected tracts subsequently averaged in order to analyse the disconnection at the group level. To exclude possible effects due to the presence of damages directly associated to neurological/neuropsychological deficits, we performed disconnectome analysis on a subgroup of 16 E+ and 16 E− patients balanced for motor, tactile and proprioceptive deficits and extrapersonal neglect. The analysis revealed the most likely disconnected tracts for both groups of patients including AF, corpus callosum, CS tract, fronto-striatal tract, inferior fronto-occipital tract and SLF (Fig. 2). For comparison, see lesion overlap of the selective cohort of patients shown in Fig. 1A.

The averaged disconnectome maps estimated starting from the lesions observed in the large cohort of patients are reported in Supplementary Fig. 3.

**Figure 3:**
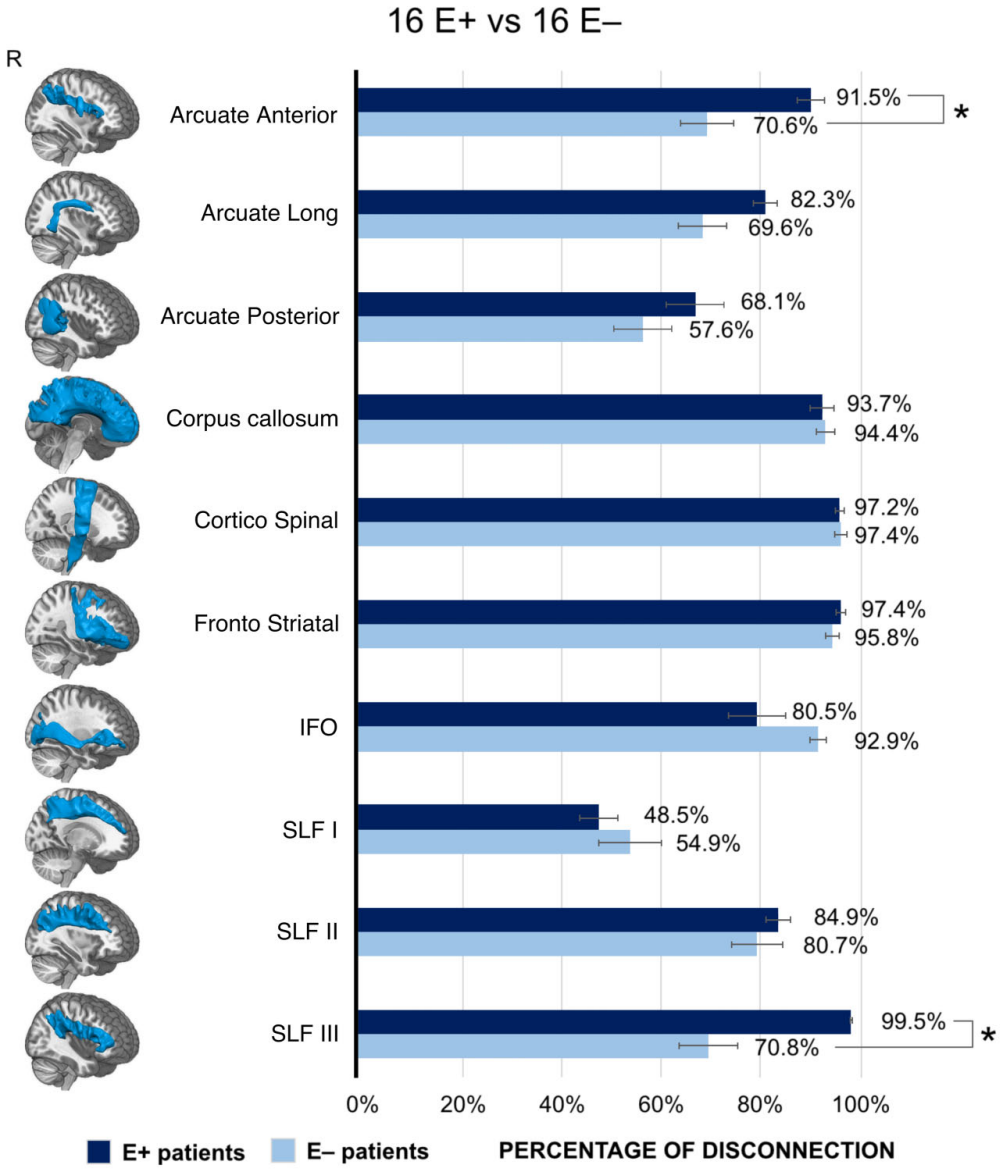
**Disconnected tracts in E+ and E− patients.** Percentage of disconnection of each of the 10 examined white matter tracts in the selective group of patients (16 E+ and 16 E−) matched for demographic and clinical variables. In order to highlight significant differences between the two groups of patients, the percentages of tract disconnection were entered, as dependent variables, a one-way MANOVA, while Group (E+ and E−) was considered as between-subject factor. Asterisks indicate significant differences for each tract between E+ and E, at *P* < 0.05, Bonferroni corrected for multiple comparisons. Each considered tract, included in the BCB toolkit,^27^ is represented in the leftmost column overlaid on a 3D render of MNI template. Error bars indicate SEM.

Figure 3 shows the percentage of disconnection in the analysis performed on the selected cohort of patients.

To estimate if the results were statistically significant, a one-way MANOVA was carried out considering the individual percentages of disconnection (calculated using Tractron tool) for the 10 most involved tracts as dependent variables and the *Group* (E+ versus E− patients) as between-subject factor.

The one-way MANOVA did not highlight a multivariate effect of Group, suggesting that between-group differences do not pertain to all tracts included in the analysis. Crucially, univariate tests (Bonferroni corrected) on each tract revealed that between-group significant differences, with E+ patients showing higher disconnection percentages relative to E− patients, are present only in the anterior segment of the AF [*F*(1,30) = 5.36; *P* = 0.03; E+ = 92%; E− = 71%] and SLF III [*F*(1,30) = 10.37; *P* = 0.003; E+ = 100%; E− = 71%].

The results of the Probit regression showed that only a disconnection of the anterior segment of AF (*B*=−0.72; *P* = 0.028) and of SLF III (*B*=−0.90; *P* = 0.003) significantly predicted the group categorization (i.e. as E+ or E−), with the disconnection of SLF III predicting the group categorization to a better extent (*R*^2^ = 0.26; adjusted *R*^2^ = 0.23) as compared with the anterior AF (*R*^2^ = 0.15; adjusted *R*^2^ = 0.12). See also Supplementary Table 2 for statistical details of the results of probit analysis about all the considered tracts.

The results concerning the disconnection probability estimated using lesions of the large cohort of patients are reported for completeness in Supplementary Fig. 4.

### DTI tractography results

**Figure 4:**
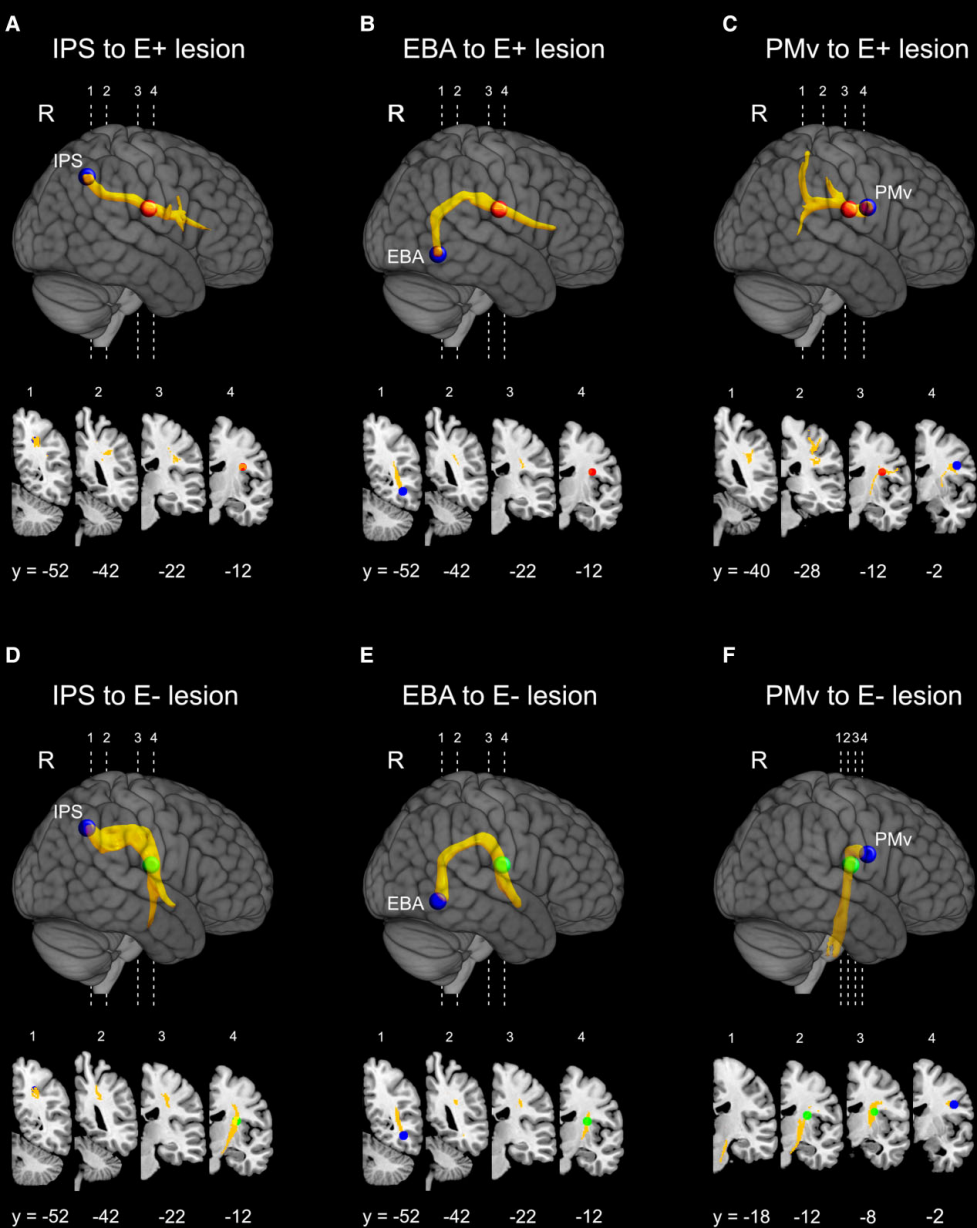
**Probabilistic tractography results.** Significant normalized and thresholded white matter tracts originating from each of the three cortical seed regions (blue coloured spheres). Waypoint masks (E+ waypoint: red coloured sphere; E− waypoint: green coloured sphere) were used to filter out not relevant streamlines (e.g. streamlines not part of white matter tracts passing through the patients’ lesions). 3D reconstruction of the averaged tracts, originating from IPS (**A** and **D**), EBA (**B** and **E**) and PMv (**C** and **F**) are shown projected on a lateral view of the right hemisphere into an MNI stereotaxic template. The corresponding fibre pathways for each tract are also represented in four representative right coronal sections. Each tract was thresholded at 0.01 (99% likelihood of connectivity) and their values normalized to a range of 0–100.000. Numbers below the sections indicate *y*-coordinates in MNI space.

Figure 4 shows the probabilistic distribution of white matter tracts connecting each seed (blue spheres) and the lesion waypoints (red sphere corresponds to E+ patients lesions overlap, green sphere corresponds to E− lesions). Each tract is represented in yellow colour, as 3D mesh reconstruction. Each mean tract is overlaid on a 3D rendering (lateral view) of right hemisphere MNI human template and on four representative right coronal sections.

Specific tracts originating from the identified cortical seeds (IPS, PMv and EBA) were strongly connected with waypoint identified by means of E+ patients lesion overlap. Specifically, both parietal and premotor seeds were connected via a route passing through the lesioned area, along the AF/SLF of all 16 subjects. The topography of the specific tract reconstructed using the IPS seed (Fig. 4A) shows a specific connection between inferior parietal areas and PMv cortex extended to the IFG sector. The temporal node (EBA) was connected via a strong ventral pathway (Fig. 4B) running through the E+ lesion overlap to the PMv cortex and the IFG sector. From the PMv cortex seed, the route passing via the lesion overlap in E+ patients was consistently represented (Fig. 4C), connecting this ventral sector of premotor cortex with both the IPS and SPL also including the primary (SI) and the secondary (SII) somatosensory cortices. Interestingly, the tracts originated from the IPS and EBA nodes passing through the maximum lesion overlap in patients without delusion of ownership (E−) (Fig. 4D and E), turned around the external capsule and joined the descending CS tract, not reaching; however, the PMv region. Thus, the anatomical connections among EBA, IPS and the PMv cortices appear to be consistent only when using as waypoint the lesion overlap of E+ patients. In line with this, also the tract originating from the PMv seed arches around the E− lesion overlap (Fig. 4F) and course ventrally in the white matter of corticospinal projections.

**Figure 5:**
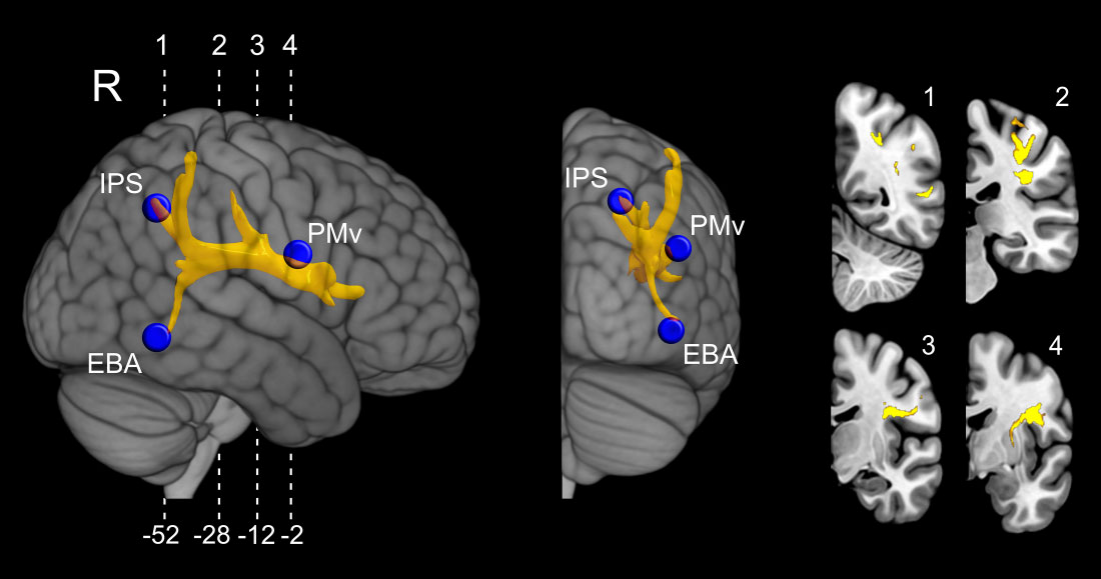
**Representation of the tracts damaged more frequently in the E+ group with respect to the E− groups.** The tract results from the regional subtraction between the normalized and thresholded fibre tracts reconstructed using the E+ and E− lesion waypoints. The resulting tract is shown from lateral right and posterior 3D views of an MNI stereotaxic template with projection on cortical surface, and in four coronal representative slices, taken at the level shown in the lateral view.

Figure 5 shows the results of the subtraction analysis between the normalized average tracts originating from PMv, IPS and EBA seeds and passing through E+ lesion versus the normalized tracts originating from the same seeds but running through the E− lesion waypoint. Thus, the resulting average tract represents, probabilistically, the most involved bundle of fibres interrupted in patients with delusion of ownership (E+). It appears that this tract connects consistently the PMv cortex not only with the intraparietal node, but also with the superior parietal lobule and primary and associative somatosensory areas, corresponding to the SLF III. Moreover, the analysis reveals the involvement of anatomical connections originating from EBA temporal node and running through the E+ lesions waypoint, corresponding to posterior AF, indicating that this sector of the reconstructed tract was more largely damaged in E+ patients as compared with E− ones. Note that previous studies^53^ demonstrated the possibility to distinguish the classical AF component (posterior temporal sector) from the SLF III, which runs parallel to this latter under the central sulcus in the fronto-parietal white matter.

### Case series results

**Figure 6:**
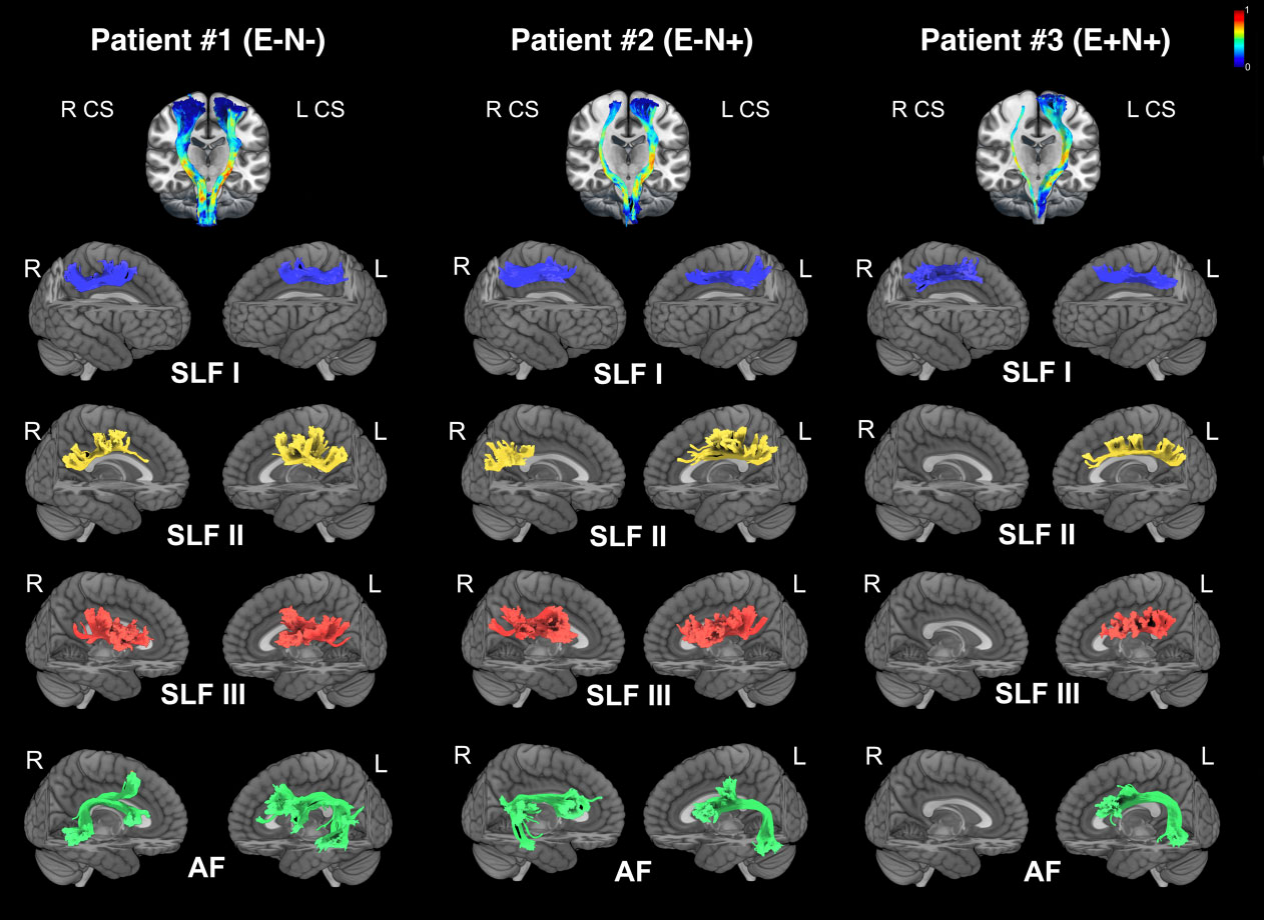
**Probabilistic *‘in vivo*’ tractography in the three patients E−N−, E−N+ and E+N+.** The analysis includes the tractography of CS tract, SLF branches and AF (both hemispheres). CS tracts are shown on a 3D coronal view on an MNI template. Colour scale indicates the FA value for both right and left CS tracts. Tractography of the SLF branches and the AF (both hemispheres) of each patient are shown on a lateral render of MNI template.

Figure 6 shows the reconstruction of the CS tract, the SLF (I, II, III) and the AF of both left (spared) and right (damaged) hemispheres in three patients (see section ‘Materials and methods’). Tractography reconstruction showed intact CS, SLF I, II, III and AF in the left hemisphere for all patients. Regarding the right hemisphere, in Patient #1 (E−N−), no significant damage of the parieto-frontal-subcortical connections was evident. In fact, it was possible to completely reconstruct the CS, SLF I, II, III and AF tracts. In contrast, Patient #2 (E−N+) showed intact CS, SLF I, SLF III (connecting PMv to posterior parietal cortex) and AF, whereas SLF II was severely damaged. Finally, in Patient #3 (E+N+), we were able to reconstruct partially only CS and the SLF I, whereas SLF II, SLF III and the AF were completely absent.

## Discussion

In the present study, we adopted a neuropsychological perspective, thus using pathological conditions as a model to understand the normal functioning brain. In particular, we focused on a monothematic delusion of body ownership in which the brain-damaged patients misidentify other people limbs as their own and show a PE. A previous study^11^ suggested that PE could be mainly associated with a subcortical lesion leading to a disconnection syndrome. Here, we aimed to identify the specific fibre tracts whose damage leads to PE. We compared patients with (E+) and without (E−) PE, combining three different approaches. As replicated through these different methodologies, the converging results reveal that PE emerges from the disconnection between frontal, parietal and temporal areas with the AF and the third branch of the SLF as the mainly involved fibre tracts. This, in turn, leads us to propose a possible neural mechanism for the construction of a normal functioning sense of body ownership.

Starting from the disconnection analysis, results from the two subgroups of 16 E+ and 16 E− patients, matched for the clinical features, shows that, when other concomitant deficits are balanced, between-group differences mainly pertain to SLF III and the anterior segment of the AF, that are significantly more disconnected in E+ as compared with E− patients (see Fig. 3). Note that it is very difficult to clearly distinguish the contribution of the anterior sector of AF and that of SLF III, since the lesion is localized at a level where the two tracts largely overlap. Nonetheless, the results of the probit regression indicate that the disconnection of SLF III predicts the group categorization to a better extent as compared with the anterior AF.

Moving to the second approach, probabilistic DTI tractography carried out on healthy subjects was used to trace cortical connections of fibre bundles that are potentially interrupted in patients, by using multiple ROIs in sensorimotor areas (PMv and IPS) and high order visual areas (EBA) as seeds. In addition, lesion overlap in E+ and E− groups was used as waypoint masks to restrain tract reconstruction. When using the E− patients’ lesion overlap as a waypoint, the reconstructed tracts originating from the IPS and EBA seeds do not reach the PMv region (Fig. 4D and E). This is confirmed by the fact that when the reconstruction starts from PMv, it does not reach posterior parietal and temporal areas (Fig. 4F). These findings indicate that the temporo-parieto-premotor connection is partially spared in E− patients. Conversely, when using the lesion overlap of E+ patients as a waypoint, the analysis shows a greater involvement of the connections between temporal, parietal and premotor areas. In particular, the reconstructed tracts originated from the IPS and EBA seeds reach the PMv region and that originating from PMv reaches posterior parietal and temporal areas (Fig. 4A–C). Furthermore, the subtraction analysis between the average tract passing through E+ lesion versus that running through the E− lesion waypoint clearly shows that the most involved bundle of fibres disrupted in E+ patients connects PMv not only with the intraparietal node, but also with the SPL and primary and high order somatosensory areas (Fig. 5). Coherently, the study by Zeller *et al.*^54^ combining VLSM with probabilistic DTI, provided evidence supporting the role of PMv and its connections in mediating changes in the sense of limb ownership. On the basis of previous studies,^55^ the bundle we identified in the subtraction analysis mostly corresponds to SLF III, well known to connect the IPS and the inferior parietal lobule with PMv/IFG (including BA 44, 45, 47), largely overlapping with the anterior sector of AF. Furthermore, the present analysis reveals a higher involvement of anatomical connections originating from EBA in E+ than in E− patients, very likely corresponding to the posterior sector of the AF (Fig. 5).

Finally, the involvement of SLF III and AF in PE was further supported by *in vivo* tractography conducted in three patients, showing that in E− patients the SLF III is completely spared, and the posterior part of AF is quite preserved, while in E+ patient the right SLF III and the AF are completely absent (Fig. 6). Note that in E− and E+ with neglect there is a right damage of SLF II, which has been proposed to be associated with the emergence and chronic persistence of spatial neglect.^56–58^

Taken together, the three methodological approaches employed here converge in identifying the SLF III (together with the partially overlapped anterior AF) as the mainly involved fibre tract in E+ patients, indicating that PE emerges from the disconnection between frontal, parietal and temporal areas. SLF III is the ventral component of SLF connecting the PMv to the inferior parietal cortex, while the anterior AF connects the caudal temporal lobe with the dorsolateral prefrontal cortex.^59^ Furthermore, the probabilistic DTI tractography and the *in vivo* tractography additionally show a greater involvement in E+ than in E− patients of the posterior AF that conveys information coming from occipito-temporal areas (including EBA) to temporo-parietal regions.^60^ The disconnection of these fibre tracts, in E+ patients, raises the possibility of a ventral self-body recognition route passing through regions that are crucial for the integration of multimodal information regarding the own body. Indeed, while we usually distinguish other people’s body by vision only, for self-body recognition we might rely on the integration between visual and sensorimotor representations.^61^ We can speculate that the self-body recognition develops gradually after birth, during the continuous interactions with conspecifics, when babies start looking at either their own body or that of adults and other babies. Among these visual inputs, only those that are simultaneously associated with sensorimotor representations (i.e. originating from movement perception, position sense and tactile sensation) can be gradually recognized as the self-body and discriminated from the others’ body. Coherently, when the connectivity between regions storing visual and sensorimotor representations of the body is disrupted due to a brain damage, as in E+ cases, patients lose the ability to visually discriminate their own hand from the alien hand, even if during the clinical evaluation both are visible on the table. In these patients, the ownership judgment seems to be based only on an abstract knowledge of the body structure, so that each stimulus matching the constraints of this aprioristic body representation (e.g. a human hand aligned with the shoulder and perceived in egocentric perspective), is felt as part of the own body. Importantly, in E+ patients, position sense loss always prevents the possibility to identify the own body by using proprioceptive information (see Supplementary Fig. 1); in other words, proprioceptive deficits are always present in E+ patients. However, since also many E− patients show proprioceptive deficits, we can conclude that position sense loss is probably not sufficient for PE to occur. In the present study, when proprioceptive deficits are balanced between the two groups of E+ and E− patients, significant differences between the two groups still emerged, ruling out the claim that proprioception alone could explain our results. Furthermore, also in cases included in the third analysis, two out of three patients showed proprioceptive deficits, one with (E+) and one without (E−) PE.

Interestingly, previous models of body ownership, based on its classical experimental manipulations in healthy subjects such as the rubber hand illusion,^62^ proposed that this aprioristic knowledge of the body representation is mediated by the activity of the temporo-parietal junction (TPJ), a wide region extending over part of the inferior parietal and superior temporal cortex. It has been proposed that this brain region plays a crucial role in promoting a ‘test for fit’ to select what can be accepted as a part of the own body based on specific constraints, such as those deriving from peripersonal space, proprioception and body-related visual information.^63–65^ In E+ patients, the disruption of SLF III and both anterior and posterior AF may prevent TPJ to receive sensorimotor (from premotor and parietal regions) and visual (from occipito-temporal regions) body-related information, thus making it able to rely only on aprioristic constraints to identify the own body, as E+ patients do. Based on this evidence on pathological body ownership, describing PE as a disconnection deficit, we propose that a normal sense of body ownership emerges from the integration between the visual representation of the body, computed in occipito-temporal areas, and the sensorimotor representation of the body, stored in premotor and posterior parietal areas. Accordingly, also different lines of research, employing the RHI in healthy subjects, highlighted that the normal functioning of a visual-sensorimotor network, mainly involving EBA and PMv and their functional connectivity, is critical to construct a coherent bodily self-representation and to discriminate between self and other body parts.^45,66–68^

In conclusion, the present study shows that, in brain-damaged patients, a delusional form of body ownership such as PE can be described as a disconnection deficit, mainly involving the SLF III and the AF. This finding is consistent with the hypothesis that PE does not merely result from a damage to discrete cortical areas but, conversely, it emerges from the disconnection of multiple areas. According to the classical neuropsychological inference, these results shed light on the neuroanatomical substrate that, in the normal functioning brain, allows binding self-awareness to one’s own body.

## Supplementary Material

fcac032_Supplementary_Data

## Abbreviations

AF =: arcuate fasciculus
CS =: corticospinal
DTI =: diffusion tensor imaging
E+ =: patients with pathological embodiment
E− =: patients without pathological embodiment
EBA =: extrastriate body area
PE =: pathological embodiment
IPS =: intraparietal sulcus
PMv =: ventral premotor cortex
SLF =: superior longitudinal fasciculus
