# Correction to: Structural connectivity associated with the sense of body ownership: a diffusion tensor imaging and disconnection study in patients with bodily awareness disorder

**DOI:** 10.1093/braincomms/fcac296

**Published:** 2022-11-21

Antonino Errante, Alice Rossi Sebastiano, Settimio Ziccarelli, Valentina Bruno, Stefano Rozzi, Lorenzo Pia, Leonardo Fogassi, Francesca Garbarini, Structural connectivity associated with the sense of body ownership: a diffusion tensor imaging and disconnection study in patients with bodily awareness disorder, *Brain Communications*, Volume 4, Issue 1, 2022, fcac032, https://doi.org/10.1093/braincomms/fcac032

In the originally published version of this manuscript, there was an error in the Figure 6 legend: Patient #2 E+N− should be Patient #2 E-N+

**Figure 6:**
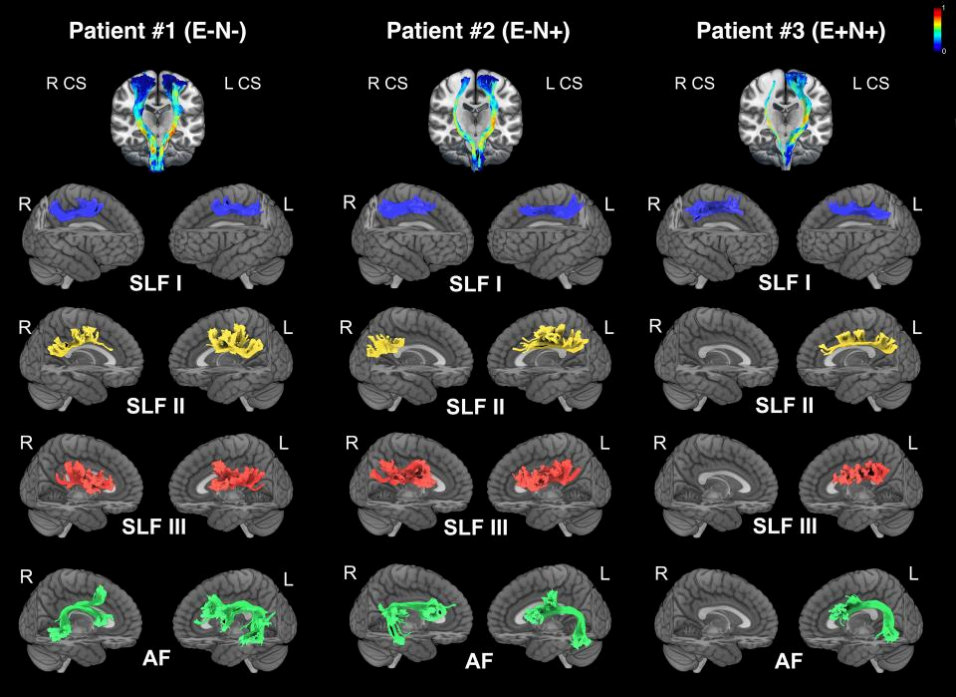
**Probabilistic ‘*in vivo*‘ tractography in the three patients E-N-, E-N+ and E+N+.** The analysis includes the tractography of CS tract, SLF branches and AF (both hemispheres). CS tracts are shown on a 3D coronal view on an MNI template. Colour scale indicates the FA value for both right and left CS tracts. Tractography of the SLF branches and the AF (both hemispheres) of each patient are shown on a lateral render of MNI template.

This error has been corrected.

