# Supplementary material for: Structural connectivity associated with the sense of body ownership: a diffusion tensor imaging and disconnection study in patients with bodily awareness disorder
Source: Brain Commun. 2022 Feb 11;4(1):fcac032. doi: 10.1093/braincomms/fcac032 (PMC8882004; doi:10.1093/braincomms/fcac032)
Supplement: fcac032_Supplementary_Data [file fcac032_supplementary_data.docx]

**Structural connectivity associated with the sense of body ownership: a DTI and disconnection study in patients with bodily awareness disorder**

**Supplementary Material**


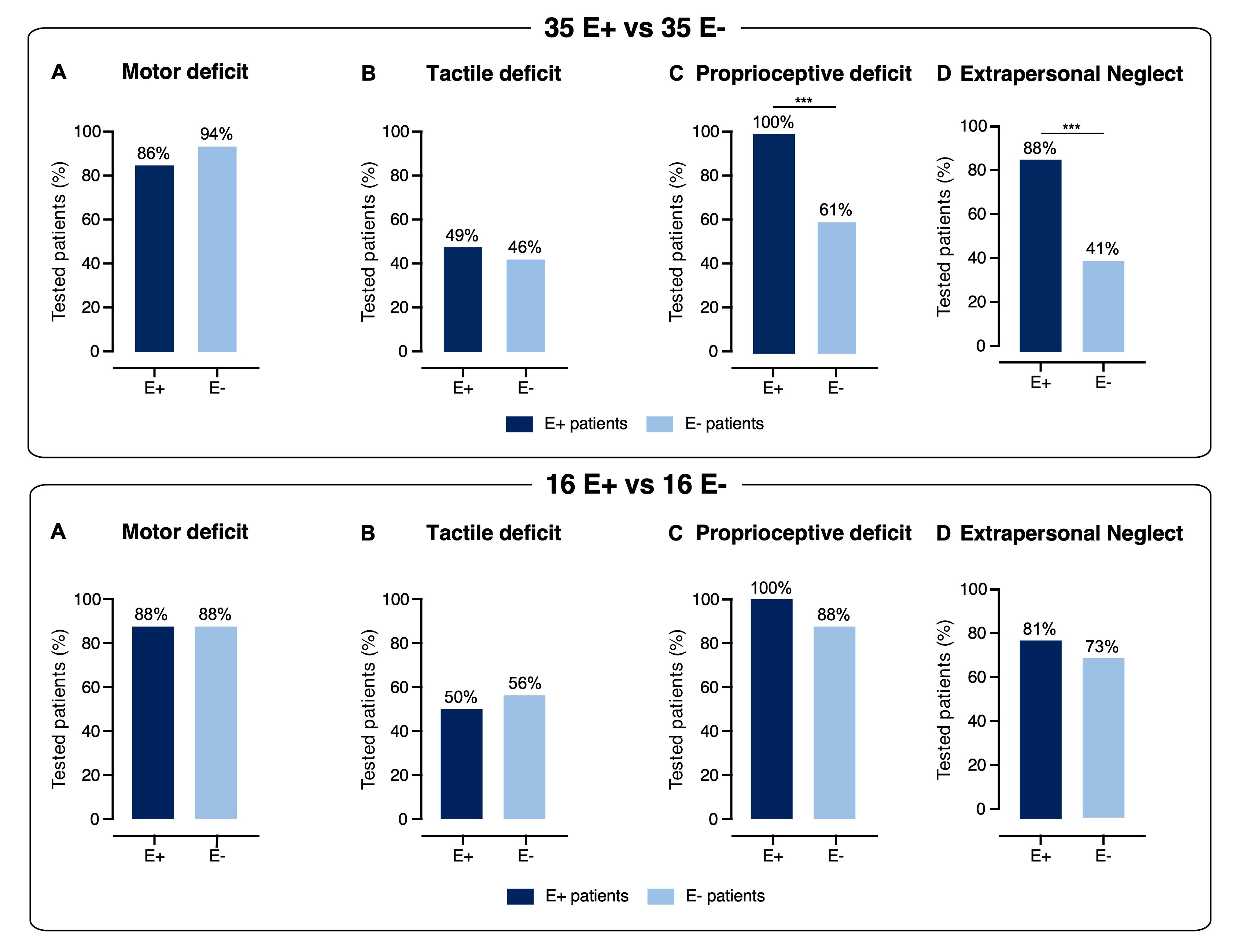


**Suppl. Fig. 1.** Patients’ clinical features. The figure represents the clinical characteristics of the large (N=70, 35 E+ vs 35 E-; top panel) and the small (N=32, 16 E+ vs 16 E-; bottom panel) sample of patients included in the disconnection analyses. Bar plots represent the percentage of E+ (dark blue) and E- (light blue) patients with motor (A), tactile (B) and proprioceptive (C) impairment and extrapersonal neglect (D). While in the large sample the percentage of E+ patients with proprioceptive deficit and extrapersonal neglect is significantly greater than the E- patients’ one (top panel; C&D), in the small sample the two groups are matched for primary and cognitive deficits (bottom panel; A,B,C,D). *** = p < 0.0005


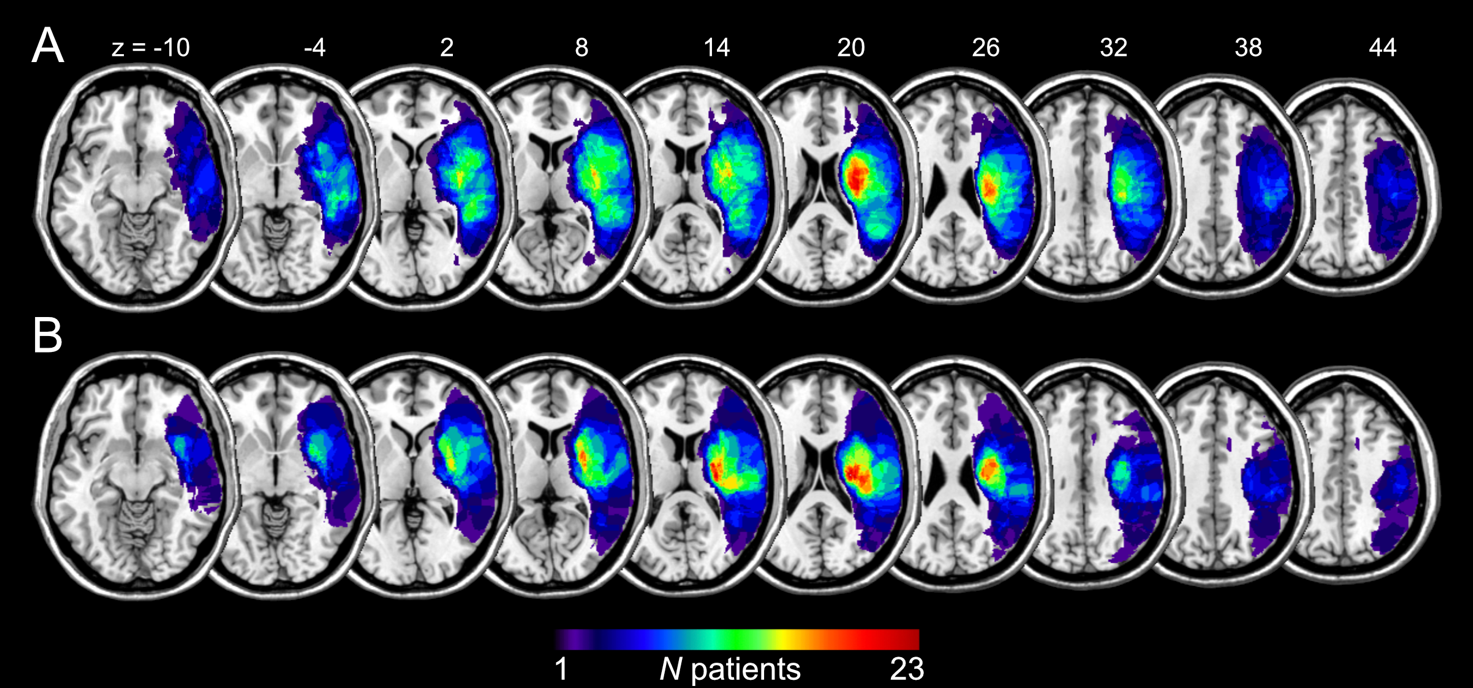


**Suppl. Fig. 2** Brain lesions overlap topography of all 35 E+ (A) and of all 35 E- (B) patients. The color bars indicate the number of overlapping lesions. Numbers above the slices indicate z-coordinates in MNI space.


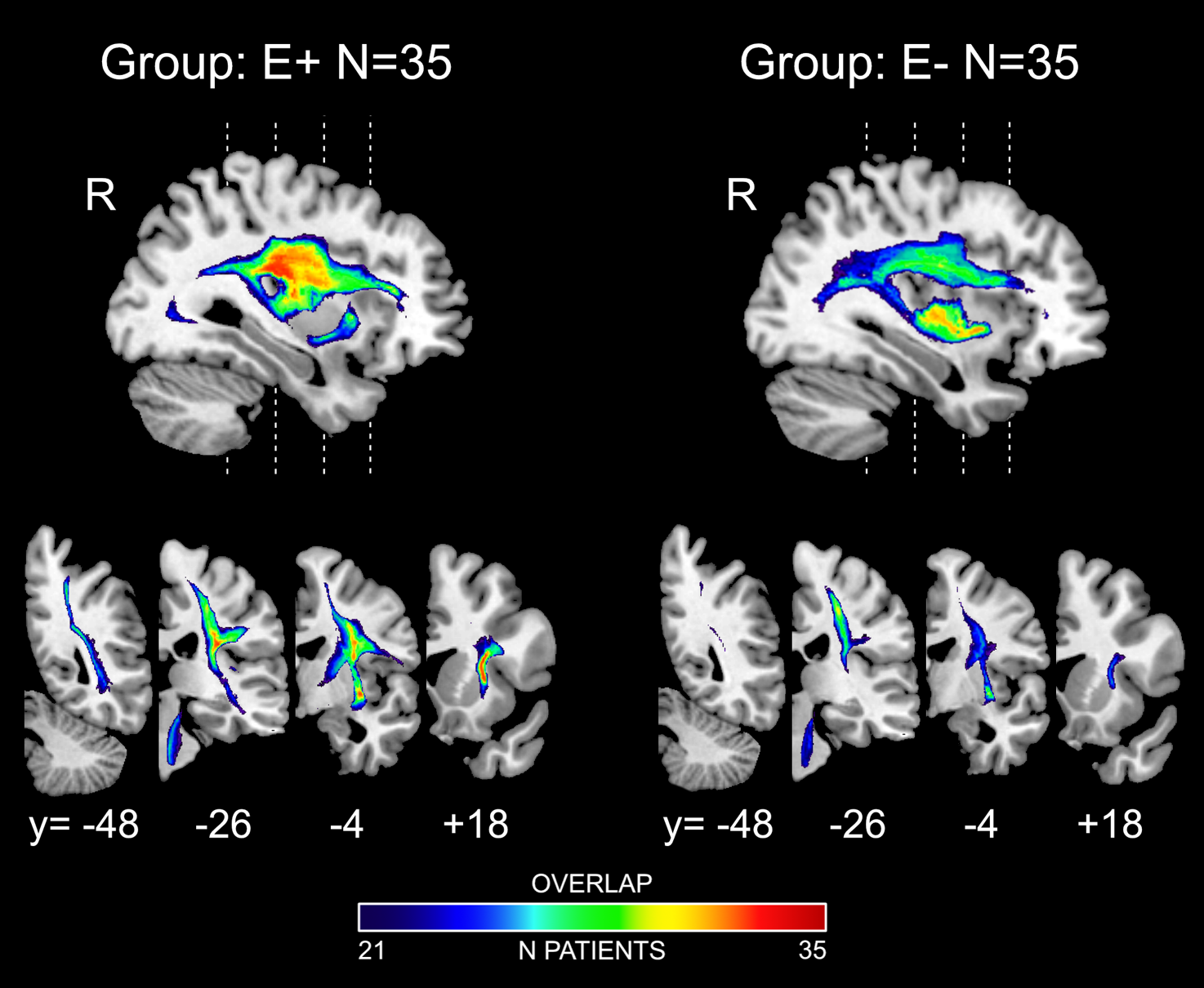


**Suppl. Fig. 3** Disconnectome maps resulting from individual lesions averaged across the large cohort of E+ and E- patients. Each map is overlaid on MNI template and presented in one medial parasagittal (x=+36 in MNI space), and four representative right coronal sections (numbers indicate y-coordinates in MNI space). The color bars indicate the number of overlapping disconnectome maps.


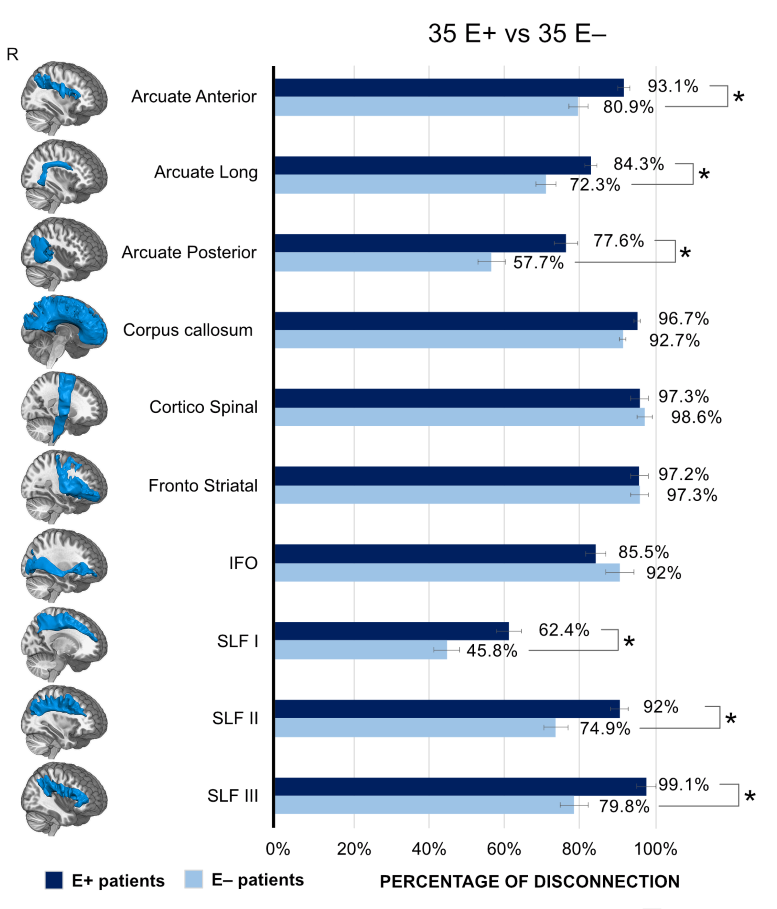


**Suppl. Fig. 4** Mean percentage of disconnection of each of the 10 examined white matter tracts in the large group of E+ patients (dark blue) and E- patients (light blue), not matched for secondary deficits. To estimate if the results were statistically significant, we ran a one-way MANOVAs on the whole group considering the individual percentages of disconnection (calculated using Tractron tool) for the 10 most involved tracts as dependent variables and the Group (E+ vs E- patients) as between subject-factor. The analysis revealed a significant multivariate effect of Group [F(10,59)= 2.83, p = 0.006]. Univariate tests on each tract showed that there was a between-group significant difference with E+ patients displaying higher disconnection percentages relative to E- patients in the anterior (F_(1,68)_= 5.38; *p* = 0.02; E+ = 93%; E- = 81%), long (F_(1,68)_= 6.02; *p* = 0.02; E+ = 84%; E- = 72%) and posterior (F_(1,68)_= 6.33; *p* = 0.01; E+ = 78%; E- = 58%) segment of the AF, as well as the SLF I (F_(1,68)_= 4.57; *p* = 0.04; E+ = 62%; E- = 46%), SLF II (F_(1,68)_= 8.23; *p* = 0.005; E+ = 92%; E- = 75%) and SLF III (F_(1,68)_= 15.88; *p* < 0.000; E+ = 99%; E- = 80%). Asterisks indicate significant differences for each tract between E+ and E, at p < 0.05, Bonferroni corrected. Each considered tract is represented in the leftmost column overlaid on a 3D render of MNI template. Error bars indicate S.E.M.

**Structural connectivity associated with the sense of body ownership: a DTI and disconnection study in patients with bodily awareness disorder**

**Suppl. Table 1**

|  | **Age** | **Gen** | **El** | **Han** | **Aet** | **Side** | **Ons** | **MoCA** | **MMSE** | **NE** | | | | |  | **AHP** | **AHA** | **Sop** | **Aso** | **UN** | **Gr** | | |  |  |
| --- | --- | --- | --- | --- | --- | --- | --- | --- | --- | --- | --- | --- | --- | --- | --- | --- | --- | --- | --- | --- | --- | --- | --- | --- | --- |
| **ID** |  |  |  |  |  |  |  |  |  | **M** | | **T** | | **PD** | **TE** |  |  |  |  |  | | **P** |  |  |  |
|  |  |  |  |  |  |  |  |  |  | **D** | **P** | **D** | **P** |  |  |  |  |  |  |  | | **E+/E-** | | |  |
| #01 | 62 | F | 8 | R | I | R | 30 | 21 | 28 | 3 | 3 | 3 | 3 | 1 | N.E. | N.A. | 1 | 0 | 0 | + | | E+ | | |  |
| #02 | 72 | F | 5 | R | I | R | N.A. | 10 | N.A. | 1 | 1 | 0 | 2 | 1 | N.E. | N.A. | 0 | N.A. | N.A. | + | | E+ | | |  |
| #03 | 77 | F | 17 | R | H | R | 35 | N.A. | 28 | 3 | 3 | 3 | 3 | 1 | N.E. | 0 | 0 | 0 | 0 | - | | E+ | | |  |
| #04 | 68 | M | 13 | R | I | R | 32 | 19 | N.A. | 3 | 3 | 0 | 0 | 1 | 1 | 0 | 0 | 0 | N.A. | - | | E+ | | |  |
| #05 | 85 | F | 11 | R | I | R | 51 | 25 | 28 | 3 | 3 | 0 | 0 | N.A. | 1 | 0 | N.E. | 0 | 0 | - | | E+ | | |  |
| #06 | 64 | M | 17 | R | I | R | 50 | N.A. | 25 | 3 | 3 | 0 | 0 | 1 | 1 | 0 | N.E. | 0 | 0 | + | | E+ | | |  |
| #07 | 67 | M | 13 | R | I | R | 55 | 19 | N.A. | 3 | 3 | 0 | 0 | 1 | 1 | 0 | 0 | 0 | N.A. | + | | E+ | | |  |
| #08 | 75 | F | 12 | R | H | R | 68 | N.A. | 18 | 2 | 2 | 2 | 2 | 1 | N.E. | 0 | 1 | 1 | 0 | + | | E+ | | |  |
| #09 | 45 | M | 5 | R | H | R | 87 | N.A. | 26.2 | 3 | 3 | 3 | 3 | 1 | N.E. | 0 | 1 | 0 | 0 | + | | E+ | | |  |
| #10 | 72 | F | 5 | R | I | R | 107 | N.A. | N.A. | 1 | 0 | 0 | 0 | 1 | 1 | 0 | 0 | 0 | N.A. | + | | E+ | | |  |
| #11 | 62 | M | 11 | R | I | R | 60 | 24 | 26 | 0 | 0 | 0 | 0 | 1 | 1 | N.E. | N.E. | 0 | 0 | + | | E+ | | |  |
| #12 | 75 | M | 13 | R | H | R | 41 | 17 | N.A. | 1 | 0 | 0 | 0 | 1 | 1 | N.A. | 0 | N.A. | N.A. | - | | E+ | | |  |
| #13 | 85 | F | 8 | R | I | R | 200 | N.A. | 21.2 | 3 | 3 | 3 | 3 | 1 | N.E. | 0 | 1 | 0 | 0 | N.A. | | E+ | | |  |
| #14 | 57 | M | 14 | R | H | L | 49 | 17 | N.A. | 3 | 3 | 0 | 0 | 1 | 1 | N.A. | 0 | 0 | 0 | + | | E+ | | |  |
| #15 | 73 | M | 11 | R | I | R | 26 | N.A. | 24.4 | 3 | 3 | 3 | 3 | 1 | N.E. | 0 | 1 | 0 | 0 | + | | E+ | | |  |
| #16 | 75 | F | 8 | R | I | R | 48 | 16 | 19.7 | 3 | 3 | 0 | 0 | 1 | 1 | N.A. | N.E. | 1 | 0 | + | | E+ | | |  |
| #17 | 68 | M | 5 | R | I | R | 40 | N.A. | 25 | 3 | 3 | 3 | 3 | 1 | N.E. | 0 | 1 | 0 | 0 | + | | E+ | | |  |
| #18 | 74 | F | 5 | R | H | R | 26 | 10 | N.A. | 3 | 3 | 0 | 0 | 1 | 1 | 0 | 0 | 0 | N.A. | + | | E+ | | |  |
| #19 | 76 | F | 5 | R | I | R | 70 | N.A. | 18,7 | 0 | 0 | 0 | 0 | 1 | 1 | 0 | 0 | 0 | N.A. | + | | E+ | | |  |
| #20 | 70 | M | 3 | R | I | R | 70 | N.A. | 23 | 2 | 2 | 0 | 0 | N.A. | 0 | 0 | N.E. | 0 | 0 | + | | E+ | | |  |
| #21 | 78 | M | 8 | R | I | R | 60 | N.A. | 29 | 0 | 0 | 3 | 3 | 1 | N.E. | N.E. | 1 | 0 | 0 | + | | E+ | | |  |
| #22 | 57 | F | 13 | R | I | R | 63 | N.A. | 25 | 3 | 3 | 3 | 3 | 1 | N.E. | 0 | 0 | 0 | 0 | + | | E+ | | |  |
| #23 | 72 | M | 8 | R | I | L | 45 | 24 | 27.4 | 3 | 3 | 3 | 3 | 1 | N.E. | 0 | 0 | 0 | 0 | + | | E+ | | |  |
| #24 | 55 | M | 5 | R | I | R | 30 | N.A. | 17.9 | 3 | 3 | 2 | 2 | 1 | N.E. | 0 | 1 | 1 | 0 | + | | E+ | | |  |
| #25 | 69 | M | 8 | R | I | R | 60 | N.A. | 27 | 3 | 3 | 0 | 0 | 1 | 1 | 0 | N.E. | 0 | 0 | - | | E+ | | |  |
| #26 | 70 | F | 8 | R | I | R | 70 | N.A. | 25 | 3 | 3 | 3 | 3 | 1 | N.E. | 0 | 0 | 1 | 0 | + | | E+ | | |  |
| #27 | 75 | M | 5 | R | I | R | 34 | 18 | N.A. | 3 | 3 | 3 | 3 | N.A. | N.E. | N.A. | 1 | 0 | N.A. | + | | E+ | | |  |
| #28 | 74 | F | 5 | R | I | R | 60 | N.A. | 16 | 3 | 3 | 3 | 3 | 1 | N.E. | 0 | 0 | 0 | 0 | + | | E+ | | |  |
| #29 | 67 | M | 5 | R | I | R | 79 | N.A. | N.A. | 0 | 0 | 0 | 0 | 1 | 1 | N.E. | 0 | N.A. | N.A. | N.A. | | E+ | | |  |
| #30 | 79 | M | 5 | R | I | R | N.A. | N.A. | 19 | 0 | 0 | 0 | 0 | 1 | 1 | 0 | 0 | 0 | N.A. | + | | E+ | | |  |
| #31 | 70 | F | 5 | R | I | R | 50 | 6 | N.A. | 3 | 3 | 0 | 0 | 1 | 0 | 0 | 0 | 0 | N.A. | N.A. | | E+ | | |  |
| #32 | 50 | F | 18 | R | I | R | 40 | N.A. | 29 | 3 | 3 | 3 | 3 | 1 | N.E. | 0 | 1 | 0 | 0 | - | | E+ | | |  |
| #33 | 82 | M | 8 | R | I | R | 45 | N.A. | 27 | 3 | 3 | 2 | 2 | 1 | N.E. | 0 | 1 | 0 | 0 | + | | E+ | | |  |
| #34 | 72 | F | 5 | R | I | R | 60 | N.A. | 28 | 3 | 3 | 0 | 0 | 1 | 1 | 0 | 1 | 0 | 0 | + | | E+ | | |  |
| #35 | 75 | F | 5 | R | I | R | 40 | N.A. | 28 | 3 | 3 | 2 | 2 | 1 | N.E. | 0 | 1 | 0 | 0 | - | | E+ | | |  |
| #36 | 59 | M | 18 | R | I | R | 112 | N.A. | 28 | 1 | 1 | 0 | 0 | 1 | 1 | N.E. | N.E. | 0 | 0 | - | | E- | | |  |
| #37 | 78 | F | 5 | R | I | R | 72 | N.A. | 26 | 3 | 3 | 0 | 0 | 0 | 0 | 0 | N.E | 0 | 0 | - | | E- | | |  |
| #38 | 49 | F | 18 | R | H | R | 70 | 10 | N.A. | 2 | 3 | 0 | 0 | 1 | 1 | 0 | N.E. | 0 | 0 | N.A. | | E- | | |  |
| #39 | 53 | M | 13 | R | H | R | 43 | 26 | N.A. | 3 | 3 | 3 | 3 | 1 | N.E. | N.A. | 0 | 0 | 0 | - | | E- | | |  |
| #40 | 60 | F | 13 | R | I | R | 60 | 30 | N.A. | 1 | 1 | 0 | 0 | 1 | 1 | N.E. | N.E. | 0 | 0 | + | | E- | | |  |
| #41 | 83 | M | 3 | R | I | R | 30 | N.A. | 25.5 | 3 | 3 | 0 | 0 | 0 | 0 | 0 | N.E. | 0 | 0 | - | | E- | | |  |
| #42 | 51 | F | 13 | L | I | R | 72 | 25 | 26 | 3 | 3 | 0 | 0 | 0 | 0 | 0 | N.E. | 0 | 0 | - | | E- | | |  |
| #43 | 73 | M | 8 | R | H | R | 100 | 26 | N.A. | 3 | 3 | 2 | 0 | 0 | 0 | N.A. | 0 | N.A. | N.A. | + | | E- | | |  |
| #44 | 55 | M | 8 | R | H | R | 64 | 24 | N.A. | 3 | 3 | 0 | 0 | N.A. | 1 | N.A. | 0 | N.A. | N.A. | - | | E- | | |  |
| #45 | 66 | M | 7 | R | I | R | 57 | 24 | N.A. | 3 | 1 | 0 | 0 | 0 | 0 | 0 | 0 | 0 | 0 | + | | E- | | |  |
| #46 | 79 | F | 13 | R | I | R | 90 | N.A. | 24 | 3 | 3 | 3 | 3 | 1 | N.E. | 0 | 0 | 0 | 0 | - | | E- | | |  |
| #47 | 66 | M | 5 | R | I | R | 370 | 17 | N.A. | 3 | 3 | 0 | 0 | 1 | 0 | 0 | N.E. | N.A. | N.A. | + | | E- | | |  |
| #48 | 65 | F | 8 | R | I | R | 40 | 24 | 28 | 3 | 3 | 3 | 3 | 1 | N.E. | 0 | 0 | 0 | 0 | + | | E- | | |  |
| #49 | 72 | M | 5 | R | H | R | 41 | 17 | N.A. | 0 | 0 | 3 | 2 | 1 | N.E. | 0 | 0 | 0 | N.A. | N.A. | | E- | | |  |
| #50 | 68 | M | 8 | R | I | R | 30 | N.A. | 28 | 3 | 3 | 2 | 2 | 1 | N.E. | 0 | 0 | 0 | 0 | - | | E- | | |  |
| #51 | 62 | M | 5 | R | H | R | 76 | N.A. | 28 | 3 | 3 | 0 | 0 | 0 | 0 | 0 | N.E | 0 | 0 | - | | E- | | |  |
| #52 | 69 | M | 19 | R | I | R | 91 | N.A. | 26 | 3 | 3 | 0 | 0 | 0 | 0 | 0 | N.E | 0 | 0 | - | | E- | | |  |
| #53 | 64 | M | 5 | R | I | R | 40 | N.A. | 26 | 3 | 3 | 2 | 2 | 1 | N.E. | 0 | 1 | 0 | 0 | - | | E- | | |  |
| #54 | 59 | M | 13 | R | H | R | 75 | 23 | N.A. | 3 | 3 | 3 | 3 | 1 | N.E. | N.A. | 0 | 0 | 0 | N.A. | | E- | | |  |
| #55 | 51 | F | 8 | R | H | R | 54 | 18 | N.A. | 1 | 1 | 0 | 0 | 1 | 1 | N.A. | 0 | N.A. | N.A. | + | | E- | | |  |
| #56 | 65 | M | 8 | R | I | R | 50 | N.A. | 28 | 3 | 3 | 3 | 3 | 1 | N.E. | 0 | 1 | 0 | 0 | + | | E- | | |  |
| #57 | 61 | M | 17 | R | H | R | 105 | 27 | N.A. | 2 | 2 | 3 | 3 | 1 | N.E. | N.A. | 0 | 0 | 0 | - | | E- | | |  |
| #58 | 80 | F | 8 | R | I | R | 52 | N.A. | N.A. | 3 | 3 | 3 | 3 | 1 | N.E. | 0 | 0 | 0 | N.A. | + | | E- | | |  |
| #59 | 67 | F | 10 | R | I | R | 27 | 27 | N.A. | 3 | 3 | 0 | 0 | 0 | 1 | 0 | N.E. | 0 | 0 | - | | E- | | |  |
| #60 | 37 | F | 18 | R | I | R | 50 | N.A. | 30 | 3 | 3 | 3 | 3 | 1 | N.E. | 0 | 0 | 0 | 0 | + | | E- | | |  |
| #61 | 48 | M | 13 | R | I | R | 101 | N.A. | 30 | 3 | 3 | 0 | 0 | 0 | 0 | 0 | N.E. | 0 | 0 | - | | E- | | |  |
| #62 | 63 | M | 13 | R | I | R | 97 | 24 | N.A. | 1 | 1 | 0 | 0 | 1 | 0 | N.E | N.E | 0 | 0 | - | | E- | | |  |
| #63 | 62 | F | 8 | R | I | R | 40 | N.A. | 28 | 3 | 3 | 3 | 3 | 1 | N.E. | 0 | 1 | 0 | 0 | + | | E- | | |  |
| #64 | 51 | M | 10 | R | H | L | 34 | 28 | N.A. | 0 | 0 | 0 | 0 | 0 | 1 | N.E. | 0 | N.A. | N.A. | N.A. | | E- | | |  |
| #65 | 55 | F | 18 | R | I | R | 41 | 30 | 29 | 3 | 3 | 0 | 0 | 0 | 1 | 0 | N.E. | 0 | 0 | - | | E- | | |  |
| #66 | 71 | F | 5 | R | I | R | 25 | 17 | N.A. | 3 | 3 | 0 | 0 | 0 | 1 | 0 | 0 | 0 | N.A. | N.A. | | E- | | |  |
| #67 | 75 | M | 8 | R | I | R | 69 | N.A. | N.A. | 3 | 3 | 0 | 0 | 0 | 0 | 0 | N.E. | 0 | 0 | + | | E- | | |  |
| #68 | 56 | M | 8 | R | I | R | 69 | 30 | N.A. | 3 | 3 | 3 | 3 | 1 | N.E. | N.A. | 0 | 0 | 0 | N.A. | | E- | | |  |
| #69 | 61 | M | 8 | R | H | R | 65 | 26 | N.A. | 3 | 3 | 0 | 0 | N.A. | 0 | 0 | N.E. | 0 | 0 | - | | E- | | |  |
| #70 | 68 | F | 13 | R | H | R | 38 | 27 | N.A. | 3 | 3 | 3 | 3 | 1 | N.E. | 0 | 0 | 0 | 0 | - | | E- | | |  |
| **Case Series Study** | | | | | | | | | | | | | | | | | | | | | | | | | |
| 77 | 75 | M | 17 | R | I | R | 103 | 26 | N.A. | 3 | 3 | 0 | 0 | 0 | 0 | 0 | 0 | 0 | N.A. | - | | E- | | |  |
| 56 | 67 | M | 13 | R | I | R | 55 | 19 | N.A. | 3 | 3 | 0 | 0 | 1 | 1 | 0 | 0 | 0 | N.A. | + | | E- | | |  |
| 27 | 72 | M | 13 | R | I | R | 78 | 19 | N.A. | 0 | 0 | 0 | 0 | 1 | 1 | 0 | 0 | 0 | N.A. | + | | E+ | | |  |

**Suppl. Table 1.** Demographical, clinical and neuropsychological data of the patients included in the disconnection analysis and the three patients included in the case series study. ID = patients' Identification number. Gen = Gender (M = Male, F = Female). El = Education Level (years of formal education). Han = Handedness (R = right, L = left). Aet = Aetiology (H= haemorrhage, I= ischemia). Side = Lesion side (R = right, L = Left). Ons = Onset (days between the disease and the assessment). MoCA = Montreal Cognitive Assessement (score range 0-30); MMSE = Mini Mental State Examination (score range = 0-30). NE = Neurological Examination for the contralesional upper limb (M = Motor deficits, T = Tactile deficits, D = distal, P = Proximal, score range 0-3; PD = Proprioceptive Deficits, 0 = absence, 1 = presence). TE = Tactile Extinction (0 = absence, 1 = presence). AHP = Anosognosia for Hemiplegia for the contralesional upper limb (0 = absence, 1 = presence; tested only in patients with severe motor deficits, namely score ≥ 2). AHA = Anosognosia for tactile deficits on the contralesional upper limb (0 = absence, 1 = presence; tested only in patients with severe tactile deficits, namely score ≥ 2). Sop = Somatoparaphrenia (0 = absence, 1 = presence). Aso = Asomatognosia (0 = absence, 1 = presence). UN = Unilateral Neglect (+ = presence; - = absence according to the Behavioural Inattention Test, score range 0-227, cut-off ≥ 196). Gr = Group (E+ = patients with pathological embodiment, E- = patients without the embodiment); N.E. = not executable. N.A. = not available.

| Tract | **R^2^** | **R^2^ adjusted** | **β** | **B** | **t_30_** | **p** |
| --- | --- | --- | --- | --- | --- | --- |
| Anterior arcuate fasciculus | **0.15** | **0.12** | **-0.39** | **-0.72** | **-2.32** | **0.027** |
| Long arcuate fasciculus | 0.07 | 0.04 | -0.27 | -0.59 | -1.56 | 0.12 |
| Posterior arcuate fasciculus | 0.02 | -0.01 | -0.15 | -0.23 | -0.86 | 0.40 |
| Corpus callosum | 0.001 | -0.03 | 0.03 | 0.12 | 0.16 | 0.87 |
| Corticospinal tract | 0.00 | -0.03 | 0.02 | 0.16 | 0.11 | 0.91 |
| Frontostriatal tract | 0.01 | -0.02 | -0.12 | -0.84 | -0.64 | 0.52 |
| Inferior Fronto-occipital fasciculus | 0.06 | 0.03 | 0.25 | 0.18 | 1.39 | 0.17 |
| Superior longitudinal fasciculus I | 0.01 | -0.02 | 0.11 | 0.17 | 0.58 | 0.56 |
| Superior longitudinal fasciculus II | 0.01 | -0.02 | -0.09 | -0.19 | -0.5 | 0.62 |
| Superior longitudinal fasciculus III | **0.26** | **0.23** | **-0.51** | **-0.9** | **-3.22** | **0.003** |

**Suppl. Table 2.** Results of probit regression run to verify which disconnected tract discriminates the two groups (E+/E-) in the restricted cohort of patients (balanced for secondary deficits).
